# Conference on Medical Education and on Medical Legislation of the American Medical Association

**Published:** 1911-04

**Authors:** 


### Conference on Medical Education and on Medical Legislation of the American Medical Association

THE seventh annual conference on medical education and on medical legislation of the American Medical Association was held in Chicago, March 1, 2, 3, 1911, with Dr. Arthur Dean Bevan in the chair.

Dr. Bevan stated that as chairman of the council on medical education of the American Medical Association he had for seven years made a special study of medical education including the conditions in foreign countries. From this study the following conclusions may be drawn: First, medicine today is a science, just as chemistry and physics are sciences. A thorough training in the sciences on which medicine is based is now essential and a thorough familiarity with disease processes in the living patient must be mastered before the student is permitted to begin practice. A close analysis of all the facts and a careful study of the results arrived at in other countries leads to the conclusion that a minimum amount of training which will make of a medical student a safe practitioner of medicine is the following: after graduating from high school he should have at least one year of training in physics, chemistry and biology; two years in the science of anatomy, physics, pharmacology and pathology; two years in the study of medicine, surgery, obstetrics and the specialties with the opportunity of studying these subjects in the dispensary and hospital and finally a year's practical training as an interne in a hospital.

### PROGRESS AND NEEDS IN MEDICAL EDUCATION.

Dr. N. P. Colwell, Chicago, secretary of the council on medical education, stated that changes for the better in medical education have been particularly rapid since 1904. Since that time, however, there has been a decrease in the number of colleges until now the number has been reduced to 129. Of the 44 colleges closed since 1907, 20 were closed outright and 24 by merging with others. Since 1904 there has also been a decided advancement in the standards of admission. Until 1904, only three or 1.9 per cent. of all medical colleges were requiring for admission more than a high school education, but now 41 or 31.8 per cent. of all colleges are requiring for admission one or more years of collegiate work, and the teaching of medicine as a whole has been placed more largely on a university basis.

### ENTRANCE EXAMINATIONS.

Dr. Thomas S. Fiske, secretary of the college entrance examination board, New York City, stated that the following manifest advantages of the examinations held by the college entrance examination board are: (1) they are uniform in subject matter; (2) they are uniformly administered; (3) they are held at many points to meet the convenience of students at one and the same time; (4) they represent the coöperative effort of a group of colleges, no one of which thereby surrenders its individuality; (5) they represent the coöperation of colleges and secondary schools in respect to a matter of vital importance to both; (6) by reason of their uniformity they aid greatly the work of the secondary schools; (7) they tend to effect a marked saving of time, money and effort in administering college admission requirements.

### SUBJECTS INCLUDED IN THE TWO YEARS OF COLLEGE WORK REQUIRED FOR ADMISSION TO MEDICAL COLLEGES.

Dr. Charles R. Bardeen, Madison, Wis., said during the two years of college work required by the majority of medical colleges which can afford to maintain high preliminary standards, about half the time should be devoted to physics, chemistry, biology and English. The other half should be divided between the study of foreign languages and one or more nontechnical studies, such as art, history, economics, civics, mathematics and philosophy. The aim of this preliminary college work should be to produce breadth of view as well as to give some understanding of scientific knowledge and methods.

### FIVE-YEAR MEDICAL COURSE.

Dr. J. G. Adami, Professor of Pathology, McGill, Montreal, stated that the objects in the development of the curriculum at McGill have been: To give a sound course in the preliminary sciences subjects; to make the courses from the third to the fifth year as practical as possible; to observe a natural sequence in the various courses; to give the maximum of hospital instruction with the freest employment of the wards and of bedside instruction.

EQUIPMENT AND INSTRUCTION OF THE LABORATORY YEARS.

Dr. E. P. Lyon, Professor of Physiology, St. Louis University School of Medicine, said the chief function of a medical school is to make good physicians. The good doctor must be an accurate observer; for training observation anatomy and its related sciences are valuable. The scientific instruction must be in the hands of professional scientists, not mere practitioners of medicine. The laboratory work should be conducted by the professors and not by cheap assistants.

INSTRUCTION AND EQUIPMENT OF THE CLINICAL YEARS.

Dr. George Blumer, dean of the medical faculty, Yale University, said that the contact of students with individual patients is the keynote of the fourth year of instruction. A system of clinical clerks is desirable. In regard to the equipment necessary there should be laboratories with sufficient microscopic and other necessary apparatus for each student; a dispensary especially equipped for teaching, with abundant material and a hospital especially equipped for teaching under competent medical school control. The greatest needs at present are proper endowments and properly paid and well trained men in the clinical years. There is also a lack of absolute control of hospitals by medical schools.

DISCUSSION.

Mr. Abraham Flexner, of New York City, said it is absolutely necessary that the function of passing on preliminary credentials or holding preliminary examinations should be taken away from the medical schools and in the case of independent medical schools and of medical departments which nominally or actually belong to universities, the entire business of passing on credentials should be handed over to the academic department.

Dr. Charles W. Dabney, President of the University of Cincinnati, advanced the general thought that in medical schools, as in engineering schools and colleges of liberal arts, we are introducing too many mechanical methods. We want educated men. We want to produce men who can observe and think and reason. Four years is a desperately short time to give a man an education. He can be given a start and can probably be trained to observe and think in that short period of time.

Dr. Victor C. Vaughan, Ann Arbor, Michigan, said we must not have cast iron rules in the teaching of medicine. We must have diversity. We must not expect any two medical schools to teach medicine in exactly the same way. He does not believe that the fifth year course is comparable with the six years combined course.

Dr. Henry A. Christian, Boston, said we have gradually in-increased the standard of medical education by increasing the preliminary preparation of the medical student. The method of pediatrics can well be applied to that phase of the subject; we should continue to feed the preliminary requirements until students gradually grow to a larger size. It may be that breast milk, or modified milk, will be best for the infant; but the infant at the present time to his mind needs a little more food and a little more growth.

SYMPOSIUM ON STATE LICENSE—VALUATION OF CREDENTIALS.

Dr. Frank B. Hiller, secretary of Missouri State Board of Health, stated that there rested on a medical licensing board a responsibility and duty three-fold in character: (1) that highest and greatest duty of safeguarding the public against the unworthy acts of the unqualified who desire to engage in the practice of medicine; (2) the duty of demanding of the institutions teaching medicine and surgery that these schools and colleges shall so measure up to the standard of equipment and teaching capacity as to permit none to leave their halls with the degree of M.D. except those who are well rounded and qualified morally and professionally to take their places in the ranks of a profession whose standards and knowledge are advancing with remarkable rapidity; (3) that of dealing justly and adequately with the applicant as he appears before such a board, an aspirant to have conferred on him the legal right to engage in the practice of the profession he has chosen.

INTERSTATE RECIPROCITY.

Dr. W. T. Gott, Secretary Indiana State Board of Medical Registration and Examinations, pointed out that the credentials issued by a board composed entirely of representatives of one system of practice should not be acceptable for a certificate through reciprocity and such a ruling would have a salutary effect in upholding the standard of educational qualifications throughout the country.

EXAMINATIONS.

Dr. William S. Fullerton, St. Paul, said the primary object of a state board examination is not to protect the physician against the unlicensed man, but to protect the public against the incompetent practitioner of medicine.

Dr. G. B. Young, Chicago, said that his experience on an examining board has led him to arrive at the conclusion that a high school certificate is very indefinite as far as giving a reliable test of the candidate's educational fitness to study or to practice medicine is concerned.

Mr. Abraham Flexner said that none of the European countries he had visited makes exclusive use of the written examination; the emphasis falls strongly on the practical features.

Dr. George H. Matson, Columbus, Ohio, said the Ohio State Board has undertaken practical examinations in physical diagnosis. They have now finished three of these examinations. Formerly, they had at their June examination from 200 to 250 applicants, but since they have established practical examinations they are having from 125 to 140. Practical examinations are a reality, not a theory.

### ATTITUDE OF THE JUDICIARY IN THE ENFORCEMENT OF MEDICAL PRACTICE ACTS.

Judge Jesse A. Baldwin, Appellate Court, Chicago, urged the profession to move forward unitedly and secure in the interests of suffering humanity the strongest and best legislation possible; continue in their efforts to compel higher standards in the profession, subordinating self-interests to the public good and he assured the profession that the attitude of the judiciary in the enforcement of legislation enacted under such conditions and to embody such purposes would be cordially sympathetic.

### WHAT SHOULD BE THE ATTITUDE OF THE STATE TOWARD THE PRACTICE OF MEDICINE?

Dr. M. L. Harris, Chicago, said that the restrictive or licensing plan of regulating the practice of medicine is fundamentally wrong in principle in that it makes the state dictatorial in a matter of personal rights which is best left to the thinking individual.

Mr. Charles R. Henderson, Professor of Sociology, University of Chicago, discussed the regulation of the practice of medicine for the public good.

Dr. A. B. Brown, Secretary Louisiana State Board of Medical Examiners, spoke of the administration of medical practice laws, and Dr. Herbert Harlan, Baltimore, spoke of the financing of the state board work.

### CONFERENCE OF THE NATIONAL LEGISLATIVE COUNCIL.

Dr. H. B. Favill, Chicago, the chairman, appointed the following committee on resolutions: Dr. E. J. McKnight, chairman, Hartford, Conn.; Dr. A. B. Brown, New Orleans; Dr. R. M. Funkhauser, St. Louis; Dr. Charles F. Whithington, Boston; and Dr. Frederick R. Green, Chicago, secretary of the council on health and public instruction, ex-officio.

The committee on resolutions presented the following report, which was adopted:

WHEREAS, The Committee on Carroll Memorial has completed its work in a highly satisfactory manner to all concerned; therefore, be it

*Resolved,* That the final report of the committee be accepted and the committee discharged; and be it further

*Resolved,* That the committee recommends that the cordial thanks of the conference be extended to Major Ireland and his committee for the efficient manner in which they have accomplished their work.

*Resolved,* That the thanks of the conference be extended to Drs. Halsey, Gay, and Bristow for their individual efforts in the consideration of medical expert testimony, and that this committee be discharged, and be it further

*Resolved,* That the Council on Health and Public Instruction be requested to establish a standing committee to give this matter thorough consideration and, if possible, to confer with a committee of the American Bar Association.

*Resolved,* That the Seventh Annual Conference of the American Medical Association on Medical Education and Medical Legislation deplores the action of Columbia University, of New York, in establishing a course of study in optometry, for the reason that opticians have never succeeded in founding an official school of optics or optometry, but have relied on the recognition or legalisation of the word optometry to make them a profession and for the further reason that the existence of such a course at Columbia University has this year been used in many states as the chief argument for the further enactment of optometry laws which are designed to permit opticians to practise medicine in a limited degree; and, be it further

*Resolved,* That a copy of these resolutions be transmitted by the secretary of the conference to the president of Columbia University of New York.

*Resolved,* That this conference reaffirms its endorsement of the model bill on vital statistics and expresses its appreciation of the interest shown by Hon. E. D. Durand, director of the census, and by Dr. Cressy L. Wilbur, chief of the division of vital statistics, in promoting the adoption of uniform vital statistic laws in the various states, and that the secretary be instructed to furnish a copy of these resolutions to the above mentioned gentlemen.

